# Analysing the evolutional and functional differentiation of four types of *Daphnia magna cryptochrome* in *Drosophila* circadian clock

**DOI:** 10.1038/s41598-019-45410-w

**Published:** 2019-06-20

**Authors:** Yohei Nitta, Sayaka Matsui, Yukine Kato, Yosuke Kaga, Kenkichi Sugimoto, Atsushi Sugie

**Affiliations:** 1 Center for Transdisciplinary Research, Niigata University, Niigata, Japan; 2 Brain Research Institute, Niigata University, Niigata, Japan; 3 Department of Cell Science, Faculty of Graduate School of Science and Technology, Niigata University, Niigata, Japan; 4 School of Medicine, Niigata University, Niigata, Japan

**Keywords:** Evolutionary genetics, Molecular evolution

## Abstract

Cryptochrome (CRY) plays an important role in the input of circadian clocks in various species, but gene copies in each species are evolutionarily divergent. Type I CRYs function as a photoreceptor molecule in the central clock, whereas type II CRYs directly regulate the transcriptional activity of clock proteins. Functions of other types of animal CRYs in the molecular clock remain unknown. The water flea *Daphnia magna* contains four *Cry* genes. However, it is still difficult to analyse these four genes. In this study, we took advantage of powerful genetic resources available from *Drosophila* to investigate evolutionary and functional differentiation of CRY proteins between the two species. We report differences in subcellular localisation of each *D. magna* CRY protein when expressed in the *Drosophila* clock neuron. Circadian rhythm behavioural experiments revealed that *D. magna* CRYs are not functionally conserved in the *Drosophila* molecular clock. These findings provide a new perspective on the evolutionary conservation of CRY, as functions of the four *D. magna* CRY proteins have diverse subcellular localisation levels. Furthermore, molecular clocks of *D. magna* have been evolutionarily differentiated from those of *Drosophila*. This study highlights the extensive functional diversity existing among species in their complement of *Cry* genes.

## Introduction

The circadian rhythm is a fundamental system for organisms, from prokaryotes to humans^1^. It is necessary to regulate a variety of animal activities, including sleep, hormone secretion, body temperature and neural activity^2–5^. External environmental cues, such as light and temperature, are crucial for entraining the rhythm^6^. Desynchronisation between internal rhythms and regular environmental changes causes a disturbance to the rhythm^7^. Furthermore, rhythm defects induce many serious health disorders^8,9^. For example, inhibiting the rhythm by artificial light at night can increase the likelihood of heart disease, diabetes, mood disorders and obesity. Despite its importance, the function and conservation of different clock genes across species remain poorly understood.

In *Drosophila melanogaster*, the molecular basis for the circadian clock has been well studied^10,11^. The *Drosophila* clock is driven by a transcriptional autoregulatory negative feedback loop, where a transcription factor, consisting of the products of *Clock* (*Clk*) and *cycle* (*cyc*) genes, promotes the expression of several clock genes, including *period* (*per*) and *timeless* (*tim*)^12^. PER and TIM, in turn, suppress their own transcription by inhibiting the transcriptional activity of CLK/CYC^13^. This negative feedback loop creates a rhythmic oscillation of these proteins. Cryptochrome (CRY), a photolyase-like UV-A/blue light receptor, is involved in the photic entrainment of the circadian rhythm^14^. Under light conditions, CRY binds to TIM in the cytosol and causes the degradation of TIM in a proteasome-dependent manner^15^. *cry* mutant flies are not able to synchronise their circadian rhythms using light^16^. Therefore, if a fly is placed under constant light conditions, a wild-type fly becomes arrhythmic, presumably due to the lack of TIM^17^, while a *cry* mutant fly displays rhythmic behaviour^16^.

The *Drosophila* CRY protein has been referred to as type I CRY. Type I CRY functions mainly as a light-responsive circadian photoreceptor in clock neurons as described above. However, unlike *Drosophila*, mammals have a second type of CRY protein (type II CRY) that directly suppresses the transcriptional activity of the CLK/BMAL1 heterodimer (the mammalian orthologue of CYC) in the nucleus in a light-independent manner^18,19^. In detail, type II CRY and PER proteins heterodimerise in the cytoplasm and move to the nucleus to suppress the transcriptional activity of the CLK/BMAL1 heterodimer, which in turn, activates the transcription of *cry* and *per* mRNA. These transcription-translation feedback loops generate the cell-autonomous molecular clock in mammals. Type I CRY also plays a role as a transcriptional repressor in the peripheral clock, such as that of the eye^20^. Some arthropods have type I and II CRY proteins, as well as two additional CRY homologues^21^. A previous study in the water flea (*Daphnia pulex*) identified four *cry* genes (*dappu-cry a*, *dappu-cry b*, *dappu-cry c* and *dappu-cry d*)^22^. Sequence similarity predicted that *dappu-cry a* and the type I CRY from *Drosophila* were homologues, as were *dappu-cry b* and the vertebrate type II CRY. *dappu-cry d* is considered to be a homologue of the CRY-DASH subfamily, which is known to repair lesions on single- and double-stranded DNA^23^. At present, there is no information regarding homology in other species with the *dappu-cry c* from *D. pulex*. Moreover, the molecular functions of and the relationships among these four CRY genes in the circadian clock are still unknown.

**Figure 1:**
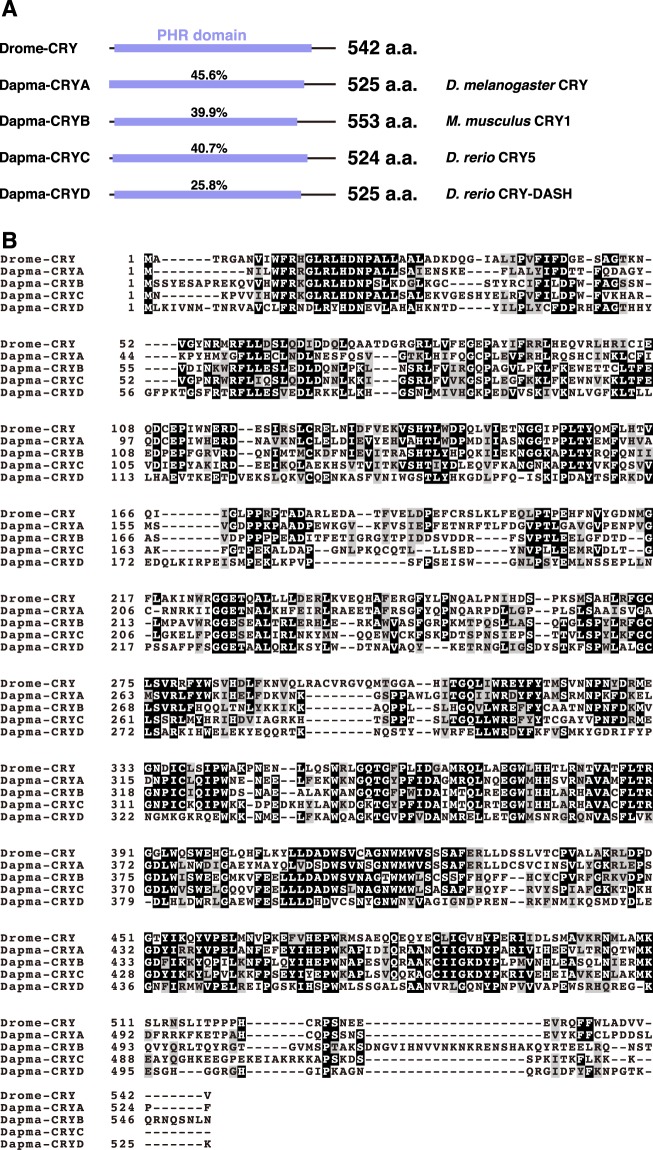
Sequence comparison of *D. melanogaster* CRY and *D. magna* CRYs. **(A**) The protein structure of *D. melanogaster* CRY (Drome-CRY) and *D. magna* CRYs (Dapma-CRYs). The percentage indicates amino acid identity of the PHR domain to that of Drome-CRY. The sequence of Drome-CRY was obtained from GenBank (accession No. AAF55469). Genes showing the highest homology to each *Dapma-cry* gene were predicted by the smartBLAST programme and positioned to the right of each *Dapma-cry* gene. **(B)** Alignment of Drome-CRY with Dapma-CRYs. Amino acids that are conserved and similar in structure are highlighted in black and grey, respectively.

To address this question, we focused on an additional water flea species. *Daphnia magna*, which is closely related to *D. pulex*, and also has all four types of *cry* genes (Fig. 1A). However, little is known about the functional differences among these genes. Therefore, we used well-established genetic tools available in *D. melanogaster*^10,11^ to establish transgenic fly lines that ectopically expressed *D. magna* CRYs. *D. magna* and *D. melanogaster* are reported to have diverged from a common ancestor approximately 666 ± 58 million years ago^24^. Using the Gal4/UAS system^25^, we examined various subcellular localisation patterns among these four CRY proteins. However, expression of *D. magna* CRYs failed to rescue the *cry* mutant flies, suggesting functional differences between *D. magna* and *D. melanogaster* CRYs.

## Results

### Sequence comparison of fly and water flea *cry* genes

To characterise the *cryptochrome* (*cry*) family in *D. magna*, we cloned and sequenced the *cry* genes (see also Methods) and designated them as *Dapma-cryA*, *Dapma-cryB, Dapma-cryC* and *Dapma-cryD*. Similar to *D. melanogaster*, all of the *D. magna* CRY proteins have an N-terminal photolyase-homologous region (PHR) domain, which contains a DNA photolyase, cryptochrome/DNA photolyase and FAD-binding domains^26^ (Fig. 1A). In addition, alignments of the amino acid sequences of Dapma-CRYs to the *D. melanogaster* CRY (Drome-CRY) revealed structural similarity between the proteins: Dapma-CRYA vs Drome-CRY, 45.2% amino acid identity/61.2% amino acid similarity; Dapma-CRYB vs Drome-CRY, 39.5% amino acid identity/56.1% amino acid similarity; Dapma-CRYC vs Drome-CRY, 41.0% amino acid identity/56.4% amino acid similarity and Dapma-CRYD vs Drome-CRY, 26.0% amino acid identity/42.7% amino acid similarity (Fig. 1B). We also compared the sequence of the CRY proteins of monarch butterfly *Danaus plexippus* (dpCRYs) with that of Drome-CRY to address the amino acid identity or similarity between the species of insect. Fly and monarch butterfly diverged from a common ancestor 295 ± 13 million years ago^27^. Comparison of the CRY proteins showed higher levels of conservation; dpCRY1^28^ vs Drome-CRY, 57.6% amino acid identity/69.2% amino acid similarity; dpCRY2^29^ vs Drome-CRY, 40.6% amino acid identity/54.9% amino acid similarity.

To find similar proteins in other species, we ran SmartBLAST (https://blast.ncbi.nlm.nih.gov/smartblast/smartBlast.cgi), using the amino acid sequences of the four Dapma-CRYs as query sequences. Dapma-CRYA showed the highest similarity to the Drome-CRY, which is type I, while Dapma-CRYB was most similar to *Mus musculus* Cryptochrome-1, which is type II (Fig. 1A). In contrast, Dapma-CRYC and D were similar to the *Danio rerio* CRY circadian clock5 (Cry5) and CRY-DASH, respectively (Fig. 1A). A previous study had indicated that Cry5 has (6–4) photolyase-like activity^30^, which is known to repair DNA damage in response to light. Therefore, Dapma-CRYC might potentially work as a photolyase. This suggestion was supported further by the cNLS mapper, which was developed to predict the classical importin-α/β pathway-specific nuclear localisation signals^31^. cNLS mapper predicted a nuclear localisation signal for only Dapma-CRYC. These results suggest functional variability among the four CRYs in the water flea. Taken together with the sequence comparison analysis between the three species and the SmartBLAST analysis, Dapma-CRYA and Drome-CRY are relatively highly conserved.

### Generation of *D. magna* CRY constructs

**Figure 2:**
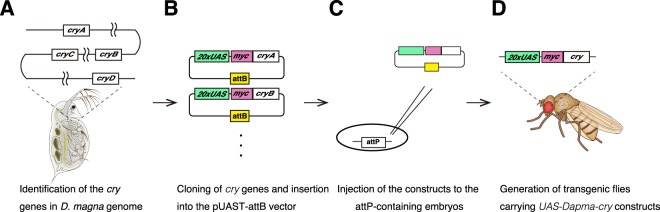
Generation of transgenic flies for the expression of the *Dapma-cryA-D*. **(A–D)** Flowchart of the *UAS-Dapma-crys* generation process. **(A)** The sequences of four *cry* genes in the *D. magna* genome were identified. **(B)** Each *cry* gene was cloned and inserted into the pUAST-attB vector. The myc-tag was placed at the N-terminal end of each *cry* cDNA. **(C)** Each construct was injected into the attP-containing embryos that express Phic31. Phic31 is an integrase that mediates the site-specific recombination at the attP site, eliminating variation in insertion sites. **(D)** Each transformant carried a *myc-Dapma-cry* downstream of the UAS sequence.

To examine whether the four Dapma-CRYs had different functions in the circadian clock and were evolutionarily conserved in other species, we utilised the Gal4/UAS system^25^ to express Dapma-CRYs in *Drosophila*. For this purpose, we generated *UAS-Dapma-cry* constructs (Fig. 2A,B). Full-length cDNAs from *Dapma-cryA*, *Dapma-cryB*, *Dapma-cryC* and *Dapma-cryD* were inserted into the pUAST-attB vector through *Xho*I and *Xba*I sites (Fig. 2B). attB is a bacterial attachment site that can efficiently integrate with a phage attachment site (attP) by the site-specific bacteriophage PhiC31 integrase^32^. A myc-tag was placed on the N-terminal end of each *Dapma-cry* (Fig. 2B), so that we could compare expression patterns of all CRYs with a single anti-myc antibody. Transgenes were inserted at identical sites using PhiC31 recombinase^32–35^. All plasmids containing attB were injected into embryos that contained the attP40 landing site on chromosome 2 and that expressed PhiC31 integrase during the embryonic stages (Fig. 2C). Transgenic flies carrying each *UAS-myc-Dapma-cry* were generated as a consequence (Fig. 2D).

### Dapma-CRYs show different subcellular localisation in the brain of *D. melanogaster*

**Figure 3:**
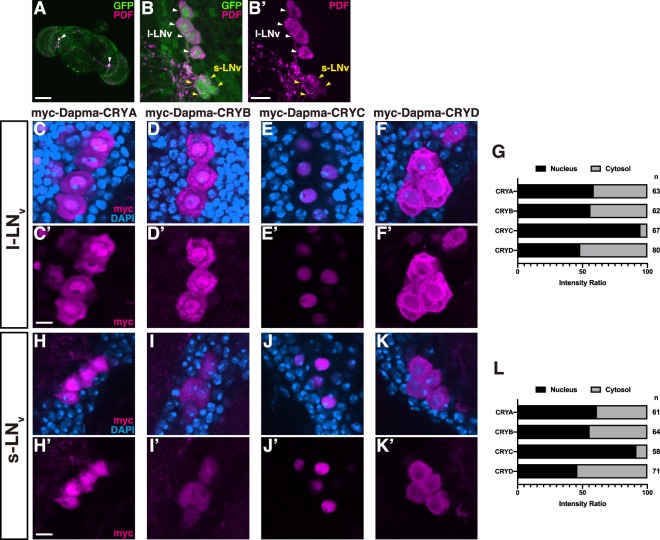
The subcellular localisation pattern of *D. magna* CRYs in l-LN_v_ and s-LN_v_. (**A**) The *tim*^62^*-Gal4* > *UAS-mCD8::GFP* expression patterns in the brain. PDF neurons were labelled using anti-PDF antibody (magenta). White arrow head denotes the cell bodies area of PDF neurons. **(B**,**B′)** Magnified view of cell bodies area of PDF neurons. The PDF positive l-LN_v_ (white arrow heads) and s-LN_v_ (yellow arrow heads) were *tim*^62^*-Gal4*-positive. (**C**–**F′**) The subcellular localisation of myc-tagged Dapma-CRYs in l-LN_v_ at ZT 1. The nuclei were stained with DAPI (cyan). Magenta signal indicates the presence of anti-myc antibody. Dapma-CRYA and Dapma-CRYB both localised to the cytosol and nucleus (**C**,**C′** and **D**,**D′**). Dapma-CRYC localised to the nucleus (**E**,**E′**). Dapma-CRYD localised to the cytosol more than the nucleus (**F**,**F′**). (**G**) The ratio of the intensity in the nuclei and the cytoplasm of myc-tagged Dapma-CRYs in l-LN_v_ neurons at ZT 1. (**H**–**K′′**) The subcellular localisation of myc-tagged Dapma-CRYs in s-LN_v_ at ZT 1. **(L)** The intensity ratio of myc-tagged Dapma-CRYs in s-LN_v_ neurons at ZT 1. n in **G** and **L** represents the number of cells quantified. Scale bars in **(A)**, 100 µm; in **(B′)**, 10 µm; in **(C′**,**H′**), 5 µm.

To characterise the intracellular molecular localisation of each Dapma-CRY in neurons, we expressed Dapma-CRYA, B, C or D under the control of *tim*^62^*-Gal4*, a clock neuron Gal4 driver^36^, since the Drome-CRY is expressed in most clock neurons^37^. Indeed, the *tim*^62^*-Gal4* driver was confirmed to be expressed in many types of clock neurons and strongly expressed in the pigment-dispersing factor (PDF)-positive neurons, l-LN_v_ and s-LN_v_ (Fig. 3A,B′), since Drome-CRY is also expressed in the l-LN_v_ and s-LN_v_^37–39^. We found that Dapma-CRYA, B and D were present in both the cytosol and nucleus of l-LN_v_ and s-LN_v_ (l-LN_v_, Fig. 3C,C′, D,D′ and F,F′; s-LN_v_, Fig. 3H,H′, I,I′ and K,K′; quantified in Fig. 3G,L), while Dapma-CRYC was localised strictly in the nucleus (l-LN_v_, Fig. 3E,E′; s-LN_v_, Fig. 3J,J′; quantified in Fig. 3G,L). Interestingly, Dapma-CRYD was present at a higher concentration in the cytosol than in the nucleus, showing a sharp contrast to Dapma-CRYC. A previous study had reported that the localisation pattern of Drome-CRY changes at different zeitgeber time points^40^. To confirm whether subcellular localisation patterns of Dapma-CRYs were also changed in different zeitgeber times, we observed the subcellular localisation at different time points (ZT 1, 5, 9, 13, 17 and 21). As a result, the nuclear localisation of myc-Dapma-CRYA to D did not change at each time point (Supplementary Fig. 1). A previous study had shown that the type I fly CRY was expressed broadly in the neuropil, cytosol and nucleus in the cell soma^37^.

Our results suggested that subcellular localisation in cell bodies of Dapma-CRYA in *D. melanogaster* was similar to that of its homologue in the fruit fly. Nevertheless, the intensity of Drome-CRY in the cytoplasmic area in the cell soma was stronger than that in the nucleus, similar to the relative localisation of Dapma-CRYD^37^. Dapma-CRYB localised to both the cytosol and nucleus in the cell soma, even though type II mammalian CRYs are localised predominantly in the nucleus^41,42^. Interestingly, the type II CRYs were also detected in the cytoplasmic area in the cell bodies^42^, implying that Dapma-CRYB might potentially be functionally conserved with mammalian CRYs. Furthermore, we found that Dapma-CRYC was localised primarily in the nucleus, which is consistent with the hypothesis that Dapma-CRYC has photolyase activity. Although the subcellular localisation pattern of the CRY-DASH subfamily has not been reported, it is surprising that the putative CRY-DASH homologue Dapma-CRYD localised to the cytoplasm (see the Discussion section). Taken together, our results indicate that the subcellular localisation of most Dapma-CRYs in *D. melanogaster* was similar to their putative homologue types, but the relative strength of the Dapma-CRYs signals between the cytosol and the nucleus in the cell soma was not identical to that of their homologues.

### Distinct functions between Dapma-CRYs and Drome-CRY

**Figure 4:**
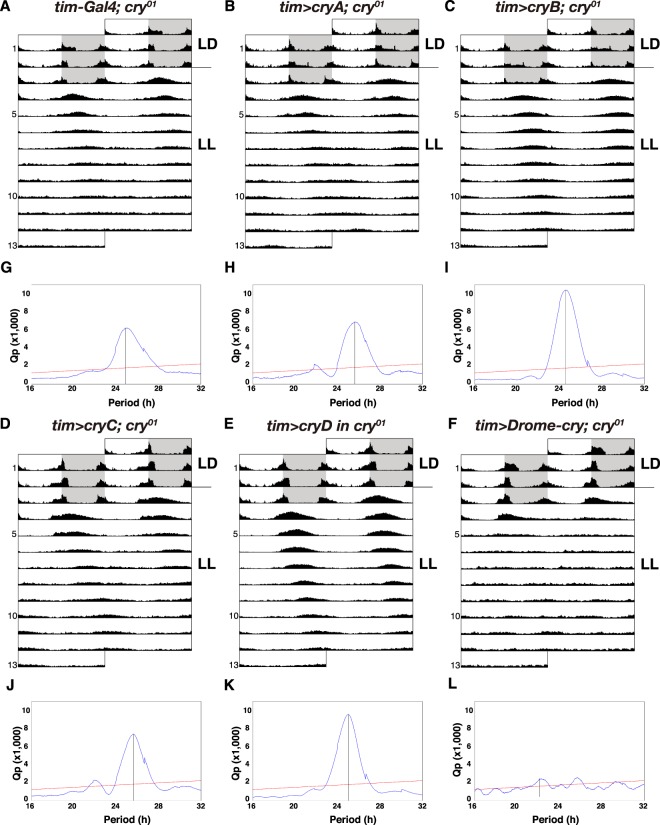
Behavioural analysis of circadian rhythm conservation between Dapma-CRYs and Drome-CRY. **(A**–**F)** Actogram showing the average locomotor activity of control (*tim*^62^*-Gal4/*+*; cry*^01^*/cry*^01^, *n* = 29, **A**), each Tim-Dapma-CRY (*tim*^62^*-gal4/UAS-Dapma-cryA; cry*^01^*/cry*^01^, *n* = 29, **B**; *tim*^62^*-gal4/UAS-Dapma-cryB; cry*^01^*/cry*^01^, *n* = 29, **C**; *tim*^62^*-gal4/UAS-Dapma-cryC; cry*^01^*/cry*^01^, *n* = 29, **D**; *tim*^62^*-gal4/UAS-Dapma-cryD; cry*^01^*/cry*^01^, *n* = 30, **E**) and Tim-Drome-CRY (*tim*^62^*-gal4/UAS-Drome-cry; cry*^01^*/cry*^01^, *n* = 29, **F**) flies. Individual male flies were entrained under a 12-h:12-h LD cycle for 3 days and then monitored under LL conditions for 10 days. Grey shading indicates the dark phase during LD. **(G–L)** The chi-squared periodogram of control **(G)**, each Tim-Dapma-CRY **(H–K)** and Tim-Drome-CRY **(L)** flies. The periodograms were obtained using records of LL conditions. The longitudinal line in each periodogram indicates the highest peak that crosses the line of significance. An oblique line in the *χ*^2^ periodogram indicates a significant level of *p* = 0.05.

Under constant light (LL) conditions, wild-type flies show an arrhythmic activity pattern, whereas *cry* mutant flies have a rhythmic locomotor activity profile because they lack photic entrainment (Fig. 4A,G)^16^. To address whether the Dapma-CRY proteins showed functional differentiation and conservation in *D. melanogaster*, we tried to rescue the behavioural phenotype of the *cry* mutant by expressing Dapma-CRYs with *tim*^62^*-Gal4*. We expected that the expression of type I Dapma-CRYA in the clock neurons would rescue the rhythmic locomotor activity since Dapma-CRYA has the sequence most similar to Drome-CRY (Fig. 1A). However, neither Dapma-CRYA, CRYB, C nor D could reverse the phenotype under LL (Fig. 4B–E and H–K). Consistent with a previous study^16^, the mutant was rescued successfully by expressing Drome-CRY (Fig. 4F,L). The N-terminal myc-tagged Drome-CRY was also able to reverse the phenotype under LL^15^, indicating that the N-terminal myc-tag of Dapma-CRYs did not interfere with normal function. Note that neither the free-running period nor the entrained phase of each Dapma-CRY showed any significant difference compared to the *cry* mutant (Table 1).

**Table 1:** Free-running period and entrained phase of each genotype.

Genotype	Free-running period (hrs)	Entrained phase (hrs)	n
*tim*^62^*-Gal4/*+*; cry*^01^*/cry*^01^	25.2 ± 0.197	1.62 ± 0.142	29
*tim* ^62^ *-gal4/UAS-Dapma-cryA; cry* ^01^ */cry* ^01^	25.4 ± 0.100	2.11 ± 0.206	29
*tim* ^62^ *-gal4/UAS-Dapma-cryB; cry* ^01^ */cry* ^01^	24.7 ± 0.121	1.94 ± 0.198	29
*tim* ^62^ *-gal4/UAS-Dapma-cryC; cry* ^01^ */cry* ^01^	25.3 ± 0.177	1.49 ± 0.123	29
*tim* ^62^ *-gal4/UAS-Dapma-cryD; cry* ^01^ */cry* ^01^	24.9 ± 0.104	1.47 ± 0.0710	30
*tim* ^62^ *-gal4/UAS-Drome-cry; cry* ^01^ */cry* ^01^	arrhythmic	1.72 ± 0.101	29

The free-running period of flies in constant light was deduced from *χ*^2^ analysis. Values are shown as mean ± SEM (standard error of the mean).

**Figure 5:**
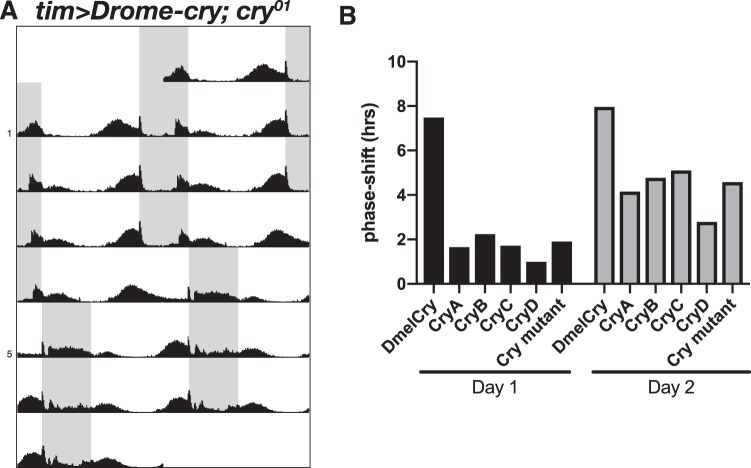
Re-entrainment shift after an 8-h LD delay. **(A)** Determination of the speed of re-entrainment between the evening activity peak of the 8-h LD phase shift. The phase of the evening activity peak was determined on the day when the LD cycle was shifted. The next day was regarded as day 1, when the activity phase shift occurred. **(B)** The speed of entrainment shifts on day 1 (left) and day 2 (right) after an 8 h LD phase delay. Tim-Drome-CRY (*tim*^62^*-gal4/UAS-Drome-cry; cry*^01^*/cry*^01^, *n* = 30) flies were phase shifted by 7 h in response to an 8 h LD shift on the first day, suggesting that these flies had almost achieved re-entrainment within one day. However, control (*tim*^62^*-Gal4/*+*; cry*^01^*/cry*^01^, *n* = 29) and each Tim-Dapma-CRY (*tim*^62^*-gal4/UAS-Dapma-cryA; cry*^01^*/cry*^01^, *n* = 30; *tim*^62^*-gal4/UAS-Dapma-cryB; cry*^01^*/cry*^01^, *n* = 30; *tim*^62^*-gal4/UAS-Dapma-cryC; cry*^01^*/cry*^01^, *n* = 30; *tim*^62^*-gal4/UAS-Dapma-cryD; cry*^01^*/cry*^01^, *n* = 30) were phase-shifted by 2 h in response to an 8-h LD shift.

To further confirm the functional conservation of Dapma-CRYs in the fly molecular clock, we investigated entrainability to light by performing re-entrainment experiments. A previous study had reported that a *cry* mutant displayed slow re-entrainment to environmental LD cycles, and that this deficit was rescued by CRY expression in CRY-positive neurons^43^. To carry out these experiments, we subjected the flies to 16/8 LD cycles and evaluated the speed of re-entrainment as previously described^43,44^. As a result, the peak of evening activity of *cry* mutant flies which expressed Drome-CRY under the control of *tim-gal4* phase-shifted by 7.5 h on day 1 after an 8-h phase shift of LD (Fig. 5A,B). This speed of re-entrainment is consistent with a previous study^43^, and it indicates that the flies showed almost complete re-entrainment to the new LD cycle within one day and that the CRY expression in TIM-positive neurons was sufficient to rescue the re-entrainment. However, *cry* mutant flies which expressed each Dapma-CRYs phase-shifted by around 2 h/d (Fig. 5B). This entrainment speed is similar to that of *cry* mutant flies, suggesting that not all Dapma-CRYs induced the TIM-degradation-dependent fast light entrainment. Taken together, the molecular functions of all Dapma-CRYs are not conserved in the core circadian clock system of *D. melanogaster*.

## Discussion

Cryptochromes are one of several core clock proteins that affect the circadian rhythm in various species. However, their subcellular localisations and functions are highly divergent^45^. Some organisms, such as water flea and monarch butterfly, have multiple types of *cry* genes in their genome, but functional differences in the circadian clock are still being determined. Our results provide novel insights into the evolution and functional differentiation of CRY proteins.

In this study, we revealed the subcellular localisation of each Dapma-CRY in the brain of *D. melanogaster* (Fig. 3): Dapma-CRYA localised to both the nucleus and cytoplasm in the cell soma; Dapma-CRYB also localised to the cytosol and nucleus; Dapma-CRYC clearly localised to the nucleus; Dapma-CRYD showed relatively strong localisation to the cytosol rather than the nucleus. Previous studies had shown that the type I CRY, putative homologues of Dapma-CRYA, localised to both the nucleus and cytoplasm^37^, whereas some of the type II CRY, putative homologues of Dapma-CRYB, also localised to both sites^42^, and *Danio rerio* Cry5, the putative homologues of Dapma-CRYC, had a nuclear localisation signal predicted by cNLS mapper. Therefore, the subcellular localisation patterns of Dapma-CRYA, Dapma-CRYB and Dapma-CRYC resemble those of their putative homologues. However, the putative CRY-DASH homologue, Dapma-CRYD, localised to the cytoplasm, which is unexpected, because the DNA-binding function of the CRY-DASH subfamily predicts that they should localise to the nucleus (Fig. 3F,F′ and K,K′). This discrepancy suggests that Dapma-CRYD might have additional functions that do not include DNA repair. Here, we analysed the Dapma-CRYs function using *Drosophila* as a protein expression system. This system is useful to evaluate the degree of conservation of the novel protein between *Drosophila* and other species. Furthermore, it is actually a powerful system to speculate on localisation patterns of unknown molecules in an animal where few genetic tools are currently available.

Our behavioural experiments showed that the Drome-CRY, but not Dapma-CRYs, rescued the rhythmic behaviour under LL conditions and the re-entrainment speed of *cry* mutant flies (Figs 4 and 5). Although we cannot rule out the possibility that the expression level of the Dapma-CRYs was insufficient to rescue the *Drome-cry* mutant phenotype, these results indicate that Dapma-CRYs cannot replace the function of the *Drosophila* CRY, which is surprising given the high sequence homology between Dapma-CRYA and Drome-CRY. Why did the ectopic expression of Dapma-CRYA in the clock neurons not rescue the *cry* mutant phenotypes? One possibility is that Dapma-CRYA failed to interact with other clock molecules in *Drosophila* due to structural differences. The PHR domain of type I CRY is responsible for phototransduction, while the C-terminal region of the protein is known to be involved in the light-dependent interactions with target proteins, including TIM^15,46,47^. Variation in the C-terminal sequences of CRY proteins generates their diversity of functions and localisation^48^. A recent study revealed that the hydrophobic FFW motif (aa 534–536) in the C-terminal region of Drome-CRY has an essential role in the correct interaction between the CRY and TIM proteins^49^. Interestingly, the hydrophobic tryptophan residue in the FFW motif is converted into cysteine, a polar amino acid residue, in Dapma-CRYA. This change could inhibit its correct interaction with the TIM protein. Future studies will be needed to reveal the interaction between Dapma-CRYA and fly TIM protein by protein-binding assays such as co-immunoprecipitation or the yeast two-hybrid system. Moreover, the binding properties of Dapma-CRYA might have diverged, because *D. pulex*, a closely relative of *D. magna*, has eight *tim* genes in its genome, whereas *D. melanogaster* has only one *tim* gene. Alternatively, water flea might have molecular clocks distinct from the fly clock. Previous studies had reported that *D. magna* has a free-running period of 28 h even under LL conditions^50,51^. However, flies show arrhythmic behaviour under these conditions due to degradation of their one TIM protein. This behavioural difference might come from the divergence of molecular clocks between water flea and fly. Our study indicates that *D. magna* and *D. melanogaster* have distinct CRY functions that affect their central molecular clocks. A previous study reported a *Drosophila cry* allele, *cry*^*m*^ ^15^, which displayed arrhythmic behaviour under LL conditions even though it was partially functional. Because the scope of the functional assays undertaken in the current paper was very limited, other functions of CRY protein, such as peripheral circadian clock, might be conserved among the two species.

The functions of the *cry* genes in *D. magna* remain unknown. Recent work has developed successful CRISPR/Cas9-mediated genome editing in *D. magna*^52,53^. These techniques will extend our understanding of the molecular functions of *D. magna* CRYs and the diversity of the circadian clock inputs.

## Methods

### Fly strains

Flies were maintained at 25 °C on standard fly food. *tim*^62^*-Gal4*^36^ (BL7126) and *UAS-mcd8::GFP*^54^ (BL32194) were obtained from the Bloomington Drosophila Stock Center. The *cry*^01^ mutant^55^, *UAS-cry*^56^ and *UAS-myc-cry*^15^ were gifts from J. Hall (Brandeis University, Waltham, MA, USA), M. Rosbash (Brandeis University, Waltham, MA, USA) and P. Emery (University of Massachusetts Medical School, Worcester, MA, USA), respectively.

### Immunohistochemistry and imaging

Immunohistochemistry was performed as described previously^57^. The following antibodies were used: mouse anti-myc (9B11, 1:500; Cell Signalling Technology), mouse anti-pdf (C7, 1:1000; Developmental Studies Hybridoma Bank) and anti-mouse Alexa Fluor 568 (1:400; Thermo Fisher Scientific). The specimens were mounted using Vectashield mounting medium with 4′,6-diamidino-2-phenylindole (DAPI; Vector Laboratories). Images were captured using an FV3000 (Olympus) confocal microscope and processed using Imaris software (Bitplane). For quantification of the localisation pattern of myc-DapmaCRYA-D and myc-Drome-CRY in cell bodies of l-LN_v_ or s-LN_v_, one- to four-day-old adult male flies were entrained to a 12-h:12-h light-dark cycle (LD; light: 200 lux) at 25 °C for 3 d. A total of 10–13 brains were immunostained and imaged at each time point (ZT 1, 5, 9, 13, 17 and 21). The areas of nucleus and cytoplasm were selected using freehand selection in Fiji, an open-source image analysis software^58^. Selection of the regions of interests (ROIs) was performed by experimenters who were blind with respect to the genotype. The mean fluorescence intensities of the ROIs were then calculated.

### Cloning of cryptochromes

For *D. magna cryB*, degenerate PCR primers based on the *cry b* gene of *D. pulex* (accession No. GNO_88183) were designed to clone the homologous gene in *D. magna*. Amplified fragments were ligated into the pMD20 plasmid vector and sequenced. We then performed 5′ and 3′ rapid amplification of cDNA ends to clone the entire coding sequence of *cryB* from *D. magna*. The entire coding sequence was deposited in Genbank as *cryB* (accession No. LC390207).

Genomic DNA data were searched to identify *D. magna cryA, cryC* and *cryD* sequences on http://server7.wfleabase.org/genome/Daphnia_magna/project/Daphnia_magna_genes_evg7/. Based on the sequence data, PCR primers for *cryA*, *cryC* and *cryD* were designed, and the three genes were amplified and cloned into the pTAC-2 vector (Biodynamics Laboratory Inc.).

### Generation of transgenic constructs

To clone the coding sequences of the *cry* genes of *D. magna*, cDNA was prepared. Briefly, total RNA was extracted from whole adult bodies using TRIzol reagent (Thermo Fisher Scientific). After DNase I treatment, the cDNA was transcribed using Superscript III reverse transcriptase (Thermo Fisher Scientific). The coding sequences of *cryA*, *cryB*, *cryC* and *cryD* of *D. magna* (accession Nos. LC390206, LC390207, LC390208 and LC390209) were cloned into the pTAC-2 cloning vector. To generate the UAS constructs, each *cry* gene was amplified using Primestar Max DNA polymerase (Takara Bio) with gene-specific primers (Table S1). The sp21-myc sense and anti-sense oligo DNA was dissolved in the appropriate buffer and denatured at 99 °C for 10 min and then gradually cooled to room temperature to make a double-stranded sp21-myc fragment. The sp21-myc fragment and amplified *cry* genes of *D. magna* (*Dapma-cry*s) were ligated with the NEB builder HiFi DNA assembly cloning kit (New England Biolabs Inc.) and then amplified by Primestar Max DNA polymerase. The myc-tag was placed at the N-terminal end of all constructs. The amplified fragment was ligated into the *Xho*I and *Xba*I double-digested 20 × UAS-IVS-pHTomato-P10 plasmid (kindly provided by D. Yamazaki, University of Tokyo, Japan) using the DNA assembly kit (New England Biolabs), followed by transformation in DH5alpha. DNA sequence data were retrieved and aligned using Genetyx software (Genetyx). These constructs were inserted into the attP40 landing site using site-specific integration *via* the phi-C31 system^32^. These plasmids were then injected into *yw; attP40* embryos (WellGenetics).

### Circadian rhythm behavioural assay

Behavioural assays were performed using a previously described procedure^43,59^, with some modifications. A DAM5 *Drosophila* activity monitor system (TriKinetics, Inc.) was used to record locomotor activity in 1 min bins. One- to four-day-old individual adult male flies were transferred into recording tubes containing 5% sucrose in 0.9% agar at one end. For constant light (LL) experiments, flies were entrained to a 12-h:12-h LD cycle (light: 200 lux) at 25 °C for 3 d and then monitored under constant light (LL) conditions for 10 d. For re-entrainment experiments, flies were entrained to a 16-h:8-h LD cycle (light: 200 lux) at 25 °C for 3 d. Thereafter, the flies were subjected to an 8-h delay phase-shift by extending the light phase. Activity records were analysed using ActogramJ^60^. The free-running period (τ) of locomotor rhythms was calculated by the *χ*^2^ method^61^. The entrained phase (ψ) was defined as previously described^62^.

## Supplementary information

supplementary information

## Data Availability

All data generated or analysed during this study are included in this published article. Additional raw data will be available on request.

## References

[1] Alberts, B. *et al*. *Molecular Biology of the Cell, Fifth edition*. (Garland Science, 2008).

[2] Borbély AA (1982). A two process model of sleep regulation. Hum. Neurobiol..

[3] Weitzman ED (1976). Circadian rhythms and episodic hormone secretion in man. Annu. Rev. Med..

[4] Refinetti R, Menaker M (1992). The circadian rhythm of body temperature. Physiol. Behav..

[5] Shibata S, Oomura Y, Kita H, Hattori K (1982). Circadian rhythmic changes of neuronal activity in the suprachiasmatic nucleus of the rat hypothalamic slice. Brain Res..

[6] Vitaterna MH, Takahashi JS, Turek FW (2001). Overview of circadian rhythms. Alcohol Res Health.

[7] Penev PD, Kolker DE, Zee PC, Turek FW (1998). Chronic circadian desynchronization decreases the survival of animals with cardiomyopathic heart disease. Am. J. Physiol..

[8] Preuss F (2008). Adverse effects of chronic circadian desynchronization in animals in a ‘challenging’ environment. American Journal of Physiology-Regulatory, Integrative and Comparative Physiology.

[9] Cho Y (2015). Effects of artificial light at night on human health: A literature review of observational and experimental studies applied to exposure assessment. Chronobiol. Int..

[10] Peschel N, Helfrich Förster C (2011). Setting the clock–by nature: circadian rhythm in the fruitfly *Drosophila melanogaster*. FEBS Letters.

[11] Tataroglu O, Emery P (2015). The molecular ticks of the *Drosophila* circadian clock. Curr. Opin. Insect Sci..

[12] Darlington TK (1998). Closing the Circadian Loop: CLOCK-Induced Transcription of Its Own Inhibitors *per* and *tim*. Science.

[13] Lee C, Bae K, Edery I (1999). PER and TIM inhibit the DNA binding activity of a *Drosophila* CLOCK-CYC/dBMAL1 heterodimer without disrupting formation of the heterodimer: a basis for circadian transcription. Mol. Cell. Biol..

[14] Cashmore AR, Jarillo JA, Wu YJ, Liu D (1999). Cryptochromes: blue light receptors for plants and animals. Science.

[15] Busza A, Emery-Le M, Rosbash M, Emery P (2004). Roles of the two *Drosophila* CRYPTOCHROME structural domains in circadian photoreception. Science.

[16] Emery P, Stanewsky R, Hall JC, Rosbash M (2000). A unique circadian-rhythm photoreceptor. Nature.

[17] Konopka RJ, Pittendrigh C, Orr D (1989). Reciprocal behaviour associated with altered homeostasis and photosensitivity of Drosophila clock mutants. J Neurogenet.

[18] Partch CL, Green CB, Takahashi JS (2014). Molecular architecture of the mammalian circadian clock. Trends in Cell Biology.

[19] Dardente H, Cermakian N (2007). Molecular circadian rhythms in central and peripheral clocks in mammals. Chronobiol. Int..

[20] Collins B, Mazzoni EO, Stanewsky R, Blau J (2006). Drosophila CRYPTOCHROME is a circadian transcriptional repressor. Curr. Biol..

[21] Yuan Q, Metterville D, Briscoe AD, Reppert SM (2007). Insect Cryptochromes: Gene Duplication and Loss Define Diverse Ways to Construct Insect Circadian Clocks. Mol. Biol. Evol..

[22] Tilden AR, McCoole MD, Harmon SM, Baer KN, Christie AE (2011). Genomic identification of a putative circadian system in the cladoceran crustacean *Daphnia pulex*. Comp. Biochem. Physiol. Part D: Genomics Proteomics.

[23] Schelvis JPM, Gindt YM (2017). A Review of Spectroscopic and Biophysical‐Chemical Studies of the Complex of Cyclobutane Pyrimidine Dimer Photolyase and Cryptochrome DASH with Substrate DNA. Photochem. Photobiol..

[24] Pisani D, Poling LL, Lyons-Weiler M, Hedges SB (2004). The colonization of land by animals: molecular phylogeny and divergence times among arthropods. BMC Biol..

[25] Brand AH, Perrimon N (1993). Targeted gene expression as a means of altering cell fates and generating dominant phenotypes. Development.

[26] Michael AK, Fribourgh JL, Van Gelder RN, Partch CL (2017). Animal Cryptochromes: Divergent Roles in Light Perception, Circadian Timekeeping and Beyond. Photochem. Photobiol..

[27] Hagner-Holler S, Pick C, Girgenrath S, Marden JH, Burmester T (2007). Diversity of stonefly hexamerins and implication for the evolution of insect storage proteins. Insect Biochem. Mol. Biol..

[28] Sauman I (2005). Connecting the navigational clock to sun compass input in monarch butterfly brain. Neuron.

[29] Zhu H, Yuan Q, Froy O, Casselman A, Reppert SM (2005). The two CRYs of the butterfly. Curr. Biol..

[30] Kobayashi Y (2000). Molecular analysis of zebrafish photolyase/cryptochrome family: two types of cryptochromes present in zebrafish. Genes Cells.

[31] Kosugi S, Hasebe M, Tomita M, Yanagawa H (2009). Systematic identification of cell cycle-dependent yeast nucleocytoplasmic shuttling proteins by prediction of composite motifs. Proc. Natl. Acad. Sci. USA.

[32] Bischof J, Maeda RK, Hediger M, Karch F, Basler K (2007). An optimized transgenesis system for *Drosophila* using germ-line-specific ϕC31 integrases. Proc. Natl. Acad. Sci. USA.

[33] Groth AC, Fish M, Nusse R, Calos MP (2004). Construction of transgenic Drosophila by using the site-specific integrase from phage phiC31. Genetics.

[34] Bateman JR, Lee AM, Wu C-T (2006). Site-specific transformation of Drosophila via phiC31 integrase-mediated cassette exchange. Genetics.

[35] Venken KJT, He Y, Hoskins RA, Bellen HJ (2006). P[acman]: A BAC Transgenic Platform for Targeted Insertion of Large DNA Fragments in *D. melanogaster*. Science.

[36] Kaneko M, Park JH, Cheng Y, Hardin PE, Hall JC (2000). Disruption of synaptic transmission or clock‐gene‐product oscillations in circadian pacemaker cells of *Drosophila* cause abnormal behavioral rhythms. J. Neurobiol..

[37] Yoshii T, Todo T, Wülbeck C, Stanewsky R, Helfrich Förster C (2008). Cryptochrome is present in the compound eyes and a subset of *Drosophila*’s clock neurons. J. Comp. Neurol..

[38] Benito J, Houl JH, Roman GW, Hardin PE (2008). The blue-light photoreceptor CRYPTOCHROME is expressed in a subset of circadian oscillator neurons in the Drosophila CNS. J. Biol. Rhythms.

[39] Hermann-Luibl C, Helfrich Förster C (2015). Clock network in Drosophila. Curr. Opin. Insect Sci..

[40] Schlichting M (2018). Cryptochrome Interacts With Actin and Enhances Eye-Mediated Light Sensitivity of the Circadian Clock in Drosophila melanogaster. Front. Mol. Neurosci..

[41] Kume K (1999). mCRY1 and mCRY2 are essential components of the negative limb of the circadian clock feedback loop. Cell.

[42] Thompson CL (2003). Expression of the Blue-Light Receptor Cryptochrome in the Human Retina. Invest. Ophthalmol. Vis. Sci..

[43] Yoshii T (2015). Cryptochrome-dependent and -independent circadian entrainment circuits in Drosophila. J. Neurosci..

[44] Rieger Dirk, Peschel Nicolai, Dusik Verena, Glotz Silvia, Helfrich-Förster Charlotte (2012). The Ability to Entrain to Long Photoperiods Differs between 3 Drosophila melanogaster Wild-Type Strains and Is Modified by Twilight Simulation. Journal of Biological Rhythms.

[45] Chaves I (2011). The Cryptochromes: Blue Light Photoreceptors in Plants and Animals. Annu. Rev. Plant Biol..

[46] Rosato E (2001). Light-dependent interaction between *Drosophila* CRY and the clock protein PER mediated by the carboxy terminus of CRY. Curr. Biol..

[47] Dissel S (2004). A constitutively active cryptochrome in *Drosophila melanogaster*. Nat. Neurosci..

[48] Chaves I (2006). Functional evolution of the photolyase/cryptochrome protein family: importance of the C terminus of mammalian CRY1 for circadian core oscillator performance. Mol. Cell. Biol..

[49] Vaidya AT (2013). Flavin reduction activates *Drosophila* cryptochrome. Proc. Natl. Acad. Sci. USA.

[50] Harris JE (1963). The role of endogenous rhythms in vertical migration. J. mar. biol. Ass. U.K..

[51] Ringelberg J, Servaas H (1971). A circadian rhythm in *Daphnia magna*. Oecologia.

[52] Kumagai H, Nakanishi T, Matsuura T, Kato Y, Watanabe H (2017). CRISPR/Cas-mediated knock-in via non-homologous end-joining in the crustacean *Daphnia magna*. PLOS ONE.

[53] Nakanishi T, Kato Y, Matsuura T, Watanabe H (2014). CRISPR/Cas-mediated targeted mutagenesis in *Daphnia magna*. PLOS ONE.

[54] Lee T, Luo L (1999). Mosaic analysis with a repressible cell marker for studies of gene function in neuronal morphogenesis. Neuron.

[55] Dolezelova E, Dolezel D, Hall JC (2007). Rhythm defects caused by newly engineered null mutations in Drosophila’s *cryptochrome* gene. Genetics.

[56] Emery P, So WV, Kaneko M, Hall JC, Rosbash M (1998). CRY, a *Drosophila* Clock and Light-Regulated Cryptochrome, Is a Major Contributor to Circadian Rhythm Resetting and Photosensitivity. Cell.

[57] Wu JS, Luo L (2006). A protocol for dissecting *Drosophila* melanogaster brains for live imaging or immunostaining. Nat. Protoc..

[58] Schindelin J (2012). Fiji: an open-source platform for biological-image analysis. Nat. Meth..

[59] Fogg PCM (2014). Class IIa histone deacetylases are conserved regulators of circadian function. J. Biol. Chem..

[60] Schmid B, Helfrich Förster C, Yoshii T (2011). A new ImageJ plug-in ‘ActogramJ’ for chronobiological analyses. J. Biol. Rhythms.

[61] Sokolove PG, Bushell WN (1978). The chi square periodogram: its utility for analysis of circadian rhythms. J. Theor. Biol..

[62] Matsumoto A (1994). Chronobiological analysis of a new clock mutant, Toki, in Drosophila melanogaster. J Neurogenet.

