# Supplementary material for: Analysing the evolutional and functional differentiation of four types of Daphnia magna cryptochrome in Drosophila circadian clock
Source: Sci Rep. 2019 Jun 20;9:8857. doi: 10.1038/s41598-019-45410-w (PMC6586792; doi:10.1038/s41598-019-45410-w)
Supplement: Supplementary file 1 — supplementary information [file 41598_2019_45410_MOESM1_ESM.pdf]

Analysing the evolutional and functional differentiation of four types of *Daphnia magna cryptochrome* in *Drosophila* circadian clock:

Supplementary Information

Yohei Nitta, Sayaka Matsui, Yukine Kato, Yosuke Kaga, Kenkichi Sugimoto and Atsushi Sugie

Supplementary data

Supplementary Table 1. PCR primers for UAS vector construction

**Supplemental Table I. PCR primers for UAS vector construction**	
oligo name	Sequence (5'-)
cry-A_CDS-F (in-fusion 20xUSA)	tctgaagaggacttgAATATTCTTTGGTTCCGCCG
cry-A_CDS-R (in-fusion 20xUSA)	AACGATTCATTCTAGACGATTCATTCTAGATTAGAAC
cry-B_CDS-F (in-fusion 20xUSA)	tctgaagaggacttgAGTAGCTATGAGTCTGCACC
cry-B_CDS-R (in-fusion 20xUSA)	AACGATTCATTCTAGACGATTCATTCTAGATTAGTTC
cry-C_CDS-F (in-fusion 20xUSA)	tctgaagaggacttgAATAAACCAGTAGTCATTCA
cry-C_CDS-R (in-fusion 20xUSA)	AACGATTCATTCTAGACGATTCATTCTAGATTATTTC
cry-D_CDS-F (in-fusion 20xUSA)	tctgaagaggacttgCTCAAGATTGTAAACATGACA
cry-D_CDS-R (in-fusion 20xUSA)	AACGATTCATTCTAGACGATTCATTCTAGATTATTTTT
20xUAS+sp21+myc Sense	GCGGCCGCGGCTCGAGAACTTAAAAAAAAAAATCAAAATGGAGCAAAAGCTCATTTCTGAAGAGGACTTG
20xUAS+sp21+myc AntiSense	CAAGTCCTCTTCAGAAATGAGCTTTTGCTCCATTTTGATTTTTTTTTTTAAGTTCTCGAGCCGCGGCCGC
F Primer（XhoI+xyn21	CTCGAGAACTTAAAAAAAAAAATCAAAATG

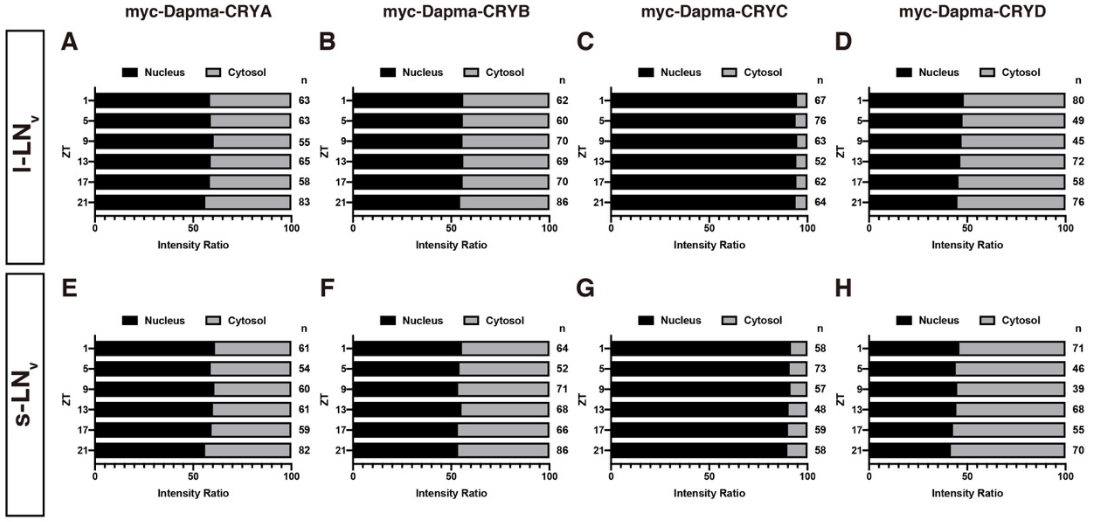

Supplementary Figure 1. Subcellular localisation of Dapma-CRYs at each time point. The ratio of the intensity in the nucleus and the cytoplasm of myc-tagged Dapma-CRYs at each zeitgeber time point (ZT 1, 5, 9, 13, 17, 21). **(A-D)** Intensity ratio of Dapma-CRYs in l-LNv. **(E-H)** Intensity ratio of Dapma-CRYs in s-LNv. n represents the number of cells quantified.
